# Maxillary sinus floor augmentation: a review of current evidence on anatomical factors and a decision tree

**DOI:** 10.1038/s41368-023-00248-x

**Published:** 2023-09-15

**Authors:** Mingyue Lyu, Dingyi Xu, Xiaohan Zhang, Quan Yuan

**Affiliations:** State Key Laboratory of Oral Diseases & National Center for Stomatology & National Clinical Research Center for Oral Diseases & West China Hospital of Stomatology, Sichuan University, Chengdu, China

**Keywords:** Surgery, Anatomy

## Abstract

Maxillary sinus floor augmentation using lateral window and crestal technique is considered as predictable methods to increase the residual bone height; however, this surgery is commonly complicated by Schneiderian membrane perforation, which is closely related to anatomical factors. This article aimed to assess anatomical factors on successful augmentation procedures. After review of the current evidence on sinus augmentation techniques, anatomical factors related to the stretching potential of Schneiderian membrane were assessed and a decision tree for the rational choice of surgical approaches was proposed. Schneiderian membrane perforation might occur when local tension exceeds its stretching potential, which is closely related to anatomical variations of the maxillary sinus. Choice of a surgical approach and clinical outcomes are influenced by the stretching potential of Schneiderian membrane. In addition to the residual bone height, clinicians should also consider the stretching potential affected by the membrane health condition, the contours of the maxillary sinus, and the presence of antral septa when evaluating the choice of surgical approaches and clinical outcomes.

## Introduction

After tooth loss, the alveolar ridge can be compromised by atrophy and pneumatization of the maxillary sinus, which might limit the success of rehabilitation.^1,2^ Maxillary sinus floor augmentation (MSFA) involves Schneiderian membrane elevation to increase the residual crestal bone height (RBH) in the posterior maxilla, thereby increasing the bone volume to install dental implants in this region, including elevation through the lateral and transcrestal approaches.^3^ Lateral window sinus augmentation, introduced by Tatum and first published by Boyne and James,^4,5^ requires visualization of a bony window in the maxillary sinus lateral wall, and suffers from post-surgical complications, high cost, and complex procedures.^6–8^ The less invasive transcrestal approach first proposed by Tatum and adapted by Summers,^5,9^ is a blind technique, with advantages such as a small wound, short operation time, and high patient satisfaction.^9^ Evidence supports the view that MSFA through both of the above approaches is effective and safe.^5,10^ With the improvement of surgical techniques and equipment, the effect of anatomical factors and the choice of surgical approaches has been continuously updated.^11–17^

**Fig. 1:**
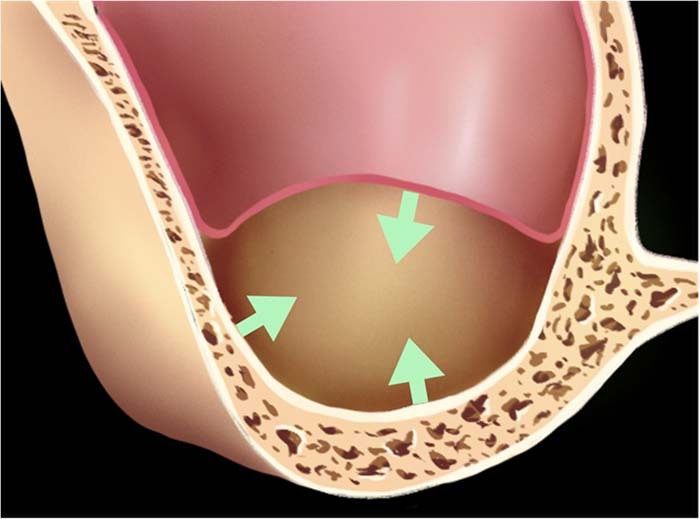
Three walls of confined space over the maxillary floor: the lateral wall of the maxillary sinus, the alveolar ridge crest, and the stretched and lifted maxillary sinus membrane

MSFA comprises the following steps: elevation of a flap, accessing the sinus cavity through a window, and Schneiderian membrane elevation above the maxillary floor and underneath the Schneider membrane to increase the alveolar bone height and create a “confined space”.^18^ Observation of this confined space from the coronal plane shown in Fig. 1, reveals that it consists of three walls: the maxillary sinus lateral wall, the crest of the alveolar ridge, and the stretched and lifted maxillary sinus membrane. Research suggested that an average tension of 7.3 N/mm³ caused sinus membrane perforation, and the stretching of the membrane can be achieved in one dimension up to 132.6% of its original size and in two dimensions up to 124.7%.^19^ In spite of the predictability of MSFA techniques, the Schneiderian membrane might be perforated when the local tension exceeds its stretching potential,^20–24^ which is also closely related to anatomical variations of this “confined space”.

This article aims to: (1) Review the impact of the above-mentioned three walls of the confined space over the maxillary floor on sinus augmentation techniques; and (2) Propose a decision tree on the choice of surgical approaches.

## Three walls of the confined space over the maxillary floor

### Residual crestal bone height

The RBH is an important factor informing the choice of surgical approach. The criteria for the minimum effective implant osseointegration height of implants have been explored, along with the range of RBH to elevate the transcrestal sinus floor.

According to the academy of Osseointegration Consensus Conference on sinus grafts (1996), elevation of the MSFA can be carried out according to the category of the classification below: Category A (RBH ≥ 10 mm): classic implant procedure; Category B (RBH ≥ 7–9 mm): osteotome technique with simultaneous placement of implants ; Category C (RBH ≥ 4–6 mm): maxillary sinus elevation with lateral access and bone graft and immediate or deferred placement of implants; Category D (RBH ≥ 1–3 mm): maxillary sinus elevation with lateral access and bone graft and deferred placement of implants.^25^ The impact of anatomical factors and the choice of surgical approaches has been continuously updated and several studies reported that the RBH did not appear to affect osteogenesis after MSFA,^12,13,26^ suggesting that more emphasis should be placed on surgical difficulty and complications, rather than osteogenesis, when considering effects of the RBH on the surgical approaches.

Although the survival rate for longer (>8 mm) implants was 0.5% higher during long-term follow-up of 16–18 months, the insertion of longer dental implants into the augmented sinus is associated with biological complications, higher morbidity, increased costs, and longer surgery, and it has been suggested that alternative treatment using shorter dental implants (≤8 mm) might be preferrable.^27,28^ The advantages of fewer complications and disadvantages of low survival rate have been compared and discussed in different length definitions of “short implants”.^29,30^ For example, it is concluded that short implants (≤6 mm) are a valid option in situations of reduced bone height to avoid possible morbidity associated with augmentation procedures; however, they reveal a higher variability and lower predictability in survival rates;^31^ while according to Group 1 ITI Consensus Report: for atrophic posterior maxilla, short implants (≤6 mm) are a promising alternative to sinus floor elevation, with comparable survival rate, less MBL (marginal bone loss) and post-surgery reactions.^32^

Subsequently, it was suggested that elevation of the transcrestal sinus floor could be extended to patients with an RBH of 4–6 mm.^33^ The crestal approach was also considered a viable technique for us in patients with an RBH ≤ 4 mm, which merits further evaluation.^34–36^ Alternatively, good clinical results were observed for posterior mandibles treated using single extra-short (4 mm) implants and Pommer et al., using multifactorial analysis of the maxillae of human cadavers, reported no significant influence of RBH on the stability of the primary implant in atrophic sinus floor.^37,38^ but initial RBH of less than 4 mm was reported to influence the success rates of implants inserted in combination with sinus floor elevation using osteotomes.^39^ Although elevation of the sinus using the transcrestal window technique in a patient with residual alveolar bone in the posterior maxilla of 1–2 mm was reported recently^11,25^ and new bone formation differences were non-significant for residual bone height ≤2 and >2 mm,^12^ the evidence was insufficient and further long-term follow-up studies were needed and data reviewed from literature suggested a higher implant survival predictability as available residual bone increases.^40^

### Lateral wall

Besides the RBH, evaluation of the lateral wall thickness should also be carried out when choosing the lateral approach. The mean maxillary lateral wall thickness has been reported as (1.98 ± 1.87) mm at the first molar and (1.21 ± 1.07) mm at the second molar.^41^ A retrospective study reported that the overall mean lateral wall thickness was (1.59 ± 0.84) mm at 4 mm coronal to the sinus floor and (1.58 ± 0.83) mm at 6 mm.^21^ Also, A. Monje et al. reported mean sinus lateral wall thickness for partial edentulous atrophic maxilla was (1.71 ± 0.12) mm, and for complete edentulous atrophic maxilla, (1.57 ± 0.07) mm.^42^

Firstly, membrane elevation requires good access and vision; however, a thick lateral wall can hinder instrument maneuverability during the lateral window sinus augmentation.^43^ Secondly, vascularization of cancellous bone is more extensive than that of cortical bone, and increased bleeding might obstruct visibility, thus prolonging surgery.^44,45^ Thirdly, membrane perforation is affected by maxillary sinus lateral wall thickness.^21,46^ At a lateral wall thickness of ≥2 mm at 4 mm coronal to the sinus floor, a perforation rate of 56.4% was observed, which decreased to 12.1% if the lateral wall thickness was ≤1 mm.^21^ And the alteration of the lateral approach sinus elevation technique is recommended if complications such as membrane perforation or bleeding are expected.^47^ Meanwhile, when accessing the antral cavity from a lateral wall of more than 2 mm, considering the vision, bleeding, and membrane perforation risk, a transcrestal approach might be a favorable alternative.

### Maxillary sinus membrane

Schneiderian membrane perforation is the most frequently reported surgical complication.^48,49^ An intact Schneiderian membrane is crucial to maintain the postoperative osteogenic space. Multiple studies reported associations between Schneiderian membrane perforation and postoperative sinusitis, bone graft infection, and implant osseointegration failure.^50–54^ Perforation might occur when the local tension exceeds the stretching potential of the Schneiderian membrane (for example, a mean perforation tension of 7.3 N·mm^−3^),^19^ which is closely related to membrane health and thickness, and anatomical characteristics, such as the maxillary sinus width and contours.^55^

#### Sinus health

Given the maxillary sinus diseases present in some patients, the application of sinus floor elevation is restricted.^25,56,57^

The presence of sinus diseases might affect the tensile potential of the Schneiderian membrane and increase the difficulty of surgery and the risk of postoperative complications.^58^ Besides, Schneiderian membrane thickened caused by inflammation might decrease elasticity and thus a reduced stretching potential. Sinusitis, polypoid (cystic) lesions, and mucosal thickening are the most frequently noted radiographical indications of sinus diseases.^59^ Small antral pseudocysts lacking clinical symptoms and slight flat thickening (>2 mm and <5 mm) are not considered as contraindications for osteotome sinus floor elevation surgery.^55^ However, pre-existing conditions that might abrogate drainage patency must be addressed.^60^

#### Schneiderian membrane thickness

An appropriate membrane thickness has an important and beneficial effects on the tensile potential of the Schneiderian membrane.^61,62^

The sinus membrane comprises a ciliated pseudostratified columnar epithelium and an average thickness = 0.5 mm.^63^ Studies of the risk factors for membrane perforation, identified that perforation was more frequent for thinner membranes.^64,65^ In a retrospective study reviewing the records of 551 patients, a thinner membrane was observed in patients who suffered membrane perforation compared with those that did not.^49^ In those that suffered perforation, the average membrane thickness was (0.84 ± 0.67) mm, whereas it was (2.65 ± 4.02) mm in the patients that did not suffer perforation.^49^

By contrast, a Schneiderian membrane thickened because of inflammation, such as from odontogenic infections, particularly apical infections,^63^ and smoking^66^ might have decreased elasticity and thus a reduced stretching potential.^67,68^ And thicker maxillary sinus membrane was indeed observed in smokers compared to non-smokers,^69,70^ and smokers (46.2%) versus nonsmokers (23.4%) presented with at least a 10% difference in rates of perforations.^71^ Certain types of irritation, e.g., allergies, are associated with mucosal thickening.^59^ Park et al. reported that perforation occurrence and preoperative sinus lesions correlated significantly,^51^ possibly because of the gelatinous structures of the pathologically thickened membranes.^72,73^

#### Sinus width

Perforation might occur when the local tension exceeds the stretching potential of Schneider’s membrane,^55^ which is closely related membrane health and thickness, and anatomical characteristics, such as the width and contours of the maxillary sinus.^74–78^

Chan et al. defined sinuses as narrow (<8 mm), average (8–10 mm), and wide (>10 mm) on the basis of a mean distance of 2.3 mm from the sinus floor, and as narrow (<14 mm), average (14–17 mm), and wide (>17 mm) on the basis of a mean distance of 15 mm from the alveolar crest.^79^ Histological analysis indicated that a narrower maxillary sinus, a sinus floor with a tapered morphology, and a higher proportion of native mineralized bone would lead to a higher level of osteogenesis after MSFA.^13^ Similarly, Stacchi C al. represented newly formed bone after transcrestal sinus floor elevation was strongly influenced by sinus width and occurred consistently only in narrow sinus cavities (sinus width <12 mm).^80^ And graft bone resorption in elevated sinus showed a positive correlation with the sinus width.^81^

Surgically, the chance of membrane perforation during elevation and the difficulty of surgery are increased by the presence of a maxillary sinus cavity with a narrow-tapered shape.^33,82^ This surgical difficulty might result from the acute angles encountered. However, the local tension increases with wider maxillary sinus floors when lifting the maxillary sinus membrane.

#### Sinus contours

Sinus contours have a vital function in procedures to elevate the sinus floor,^74–76^ and special structures such as a maxillary sinus septum and tooth roots, might increase the membrane perforation risk.^24,83,84^ Notably, when the Schneiderian membrane is raised to the same height, its different contours affect its the stretching percentage. The maxilla sinus floor could have a complicated morphology, in which the width and contours of the sinus are closely related.^85^

Sinus contours were classified into five categories by Niu et al. in 2018: Narrow tapered, tapering, ovoid, square, and irregular. Niu et al. recommended a modified lateral sinus for a narrow-tapered sinus; both lateral and transcrestal approaches for tapering and ovoid sinuses; and for irregular sinuses, a lateral sinus with a wider window or a lateral sinus with double-window floor elevation were proposed.^33^ Compared with that for a U-shaped sinus, the risk of perforation is higher for an acute angled sinus because it is more difficult to angulate the instruments.^22^ Similarly, in a review of 407 augmentation procedures, anatomical variations, including a V-shaped sinus cavity and the presence of underwood septa, were identified as potential risk factors for membrane perforation because they limit access to the antral space and obscure the surgeon’s view.^24^

#### Sinus septum

Membrane perforation risk and surgical difficulty during elevation might be increased by special structures.^24,83^ Studies have reported much higher perforation rates of MSFA in the presence of septa.^86,87^

About 20–35% of maxillary sinuses contain an antral septum.^88^ A single septum is more commonly observed than multiple septa. Shih-Cheng et al. proposed that septa could be classified as easy (E), moderate (M), or difficult (D) according to the size, orientation, number, and location of antral septa.^86^ Treatment approaches for each category have been suggested; however, antral septa complicate sinus elevation surgery.

### Assessment of membrane stretching potential: stretch-favorable type (SFT) and stretching-unfavorable type (SUT)

**Fig. 2:**
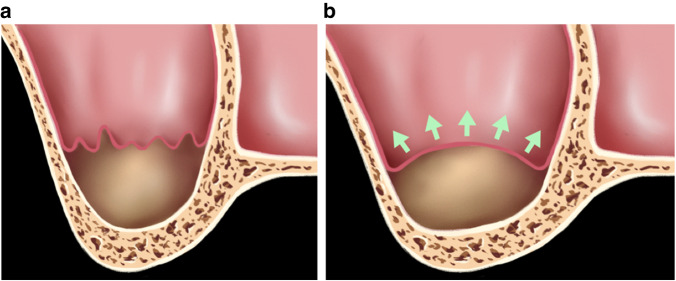
Anatomical illustrations of maxillary sinus: **a** Type A-Stretch-favorable type (SFT); **b** Type B-Stretching-unfavorable type (SUT)

Perforation might occur when the local tension exceeds the intrinsic or stretching potential of the Schneiderian membrane, which is also closely related anatomical factors, such as membrane health and the width of the maxillary sinus. The stretching potential of the Schneiderian membrane, involving the sinus width, sinus contours, sinus/membrane health, and membrane thickness, allows sinuses to be classified as the following two types (Fig. 2):

**Type A:** Stretch-favorable type (SFT). An SFT occurs when the maxillary sinus/membrane is in a healthy state; the membrane thickness is within the normal range, the maxillary contours tend to be oval; and there is no special structure, such as a maxillary sinus septum. Under these conditions, the stretching potential of the maxillary sinus membrane is relatively favorable, with relatively low surgical difficulty and risk (Fig. 2a).

**Type B:** Stretching-unfavorable type (SUT). By contrast, an SUT presents when the maxillary sinus/membrane is in a diseased state; the maxillary membrane is too thin or thickened because of inflammation; the maxillary contour tends to be wide, sharp, or angular; or there are special structures, such as a maxillary sinus septum. Under these circumstances, the stretch potential of the maxillary sinus mucosa is relatively poor, and the surgical difficulty and risks are relatively high. Table 1 lists the effects of anatomical variations on the stretching potential of Schneiderian membrane, based on the combined consideration of the sinus width, sinus contours, sinus/membrane health, and membrane thickness, which can be used to assess surgical risk and guide surgical approaches (Fig. 2b).

**Table 1:** Impact of anatomical variations on the stretching potential of the Schneiderian membrane

	Stretch-favorable type (SFT)		Stretching-unfavorable type (SUT)	
Anatomic variable	Anatomical features	Advice	Anatomical features	Advice
Sinus health condition	Health sinus	Lower perforation risk^96^	Sinus diseases	Managed with care before sinus lift procedure^59,96^
	pseudocysts in a small size without clinical symptoms	Not contraindications to surgery^55^		
Membrane thickness	Appropriate membrane thickness between 1–2 mm	Predictable sinus augmentation procedure^65^	Thinner membrane thickness of (0.84 ± 0.67) mm	Higher perforation risk compared with (2.65 ± 4.02) mm group^49^
			Thicken due to inflammation	Significant correlation between preoperative sinus lesions and perforation^51^
Sinus width and contours	Tapering or ovoid	Both lateral and transcrestal approaches are recommended^33,87^	V-shape,	Obscured visibility and limitd access to the antral space^22,24^
			Irregular	Higher perforation risk^87^
			Square	Lateral approach with a wider window^33^
Septa	Absence of septa	Lower perforation risk^86^	One or multiple septa	Higher perforation risk^24,86^

## Decision tree and clinical cases

### Decision tree

**Fig. 3:**
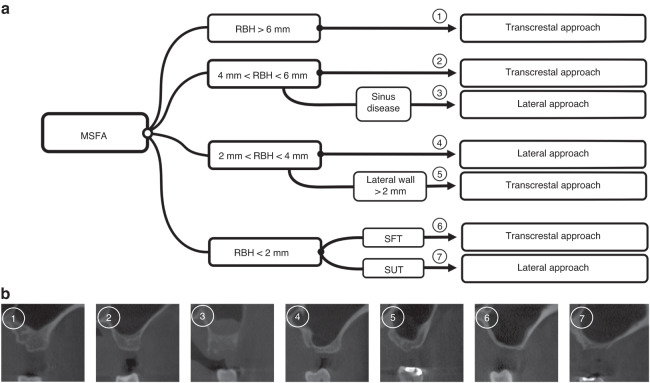
Decision tree and CBCT classification: **a** A decision tree for choice of surgical approaches; **b** Relevant 3D CBCT Classification

Ultimately, the goal of sinus elevation is to augment the available bone height to place implants, meanwhile lowering the risk of surgery. However, the success of the procedure depends on the technique chosen and treatment indications must be strictly adhered to. The RBH, lateral wall thickness, maxillary sinus contours, and the health of the Schneiderian membrane and sinus should be assessed when considering the choice of surgical approach and clinical outcomes. After a review of the literature concerning anatomical factors, and considering clinical findings, we propose the following decision tree for choosing the optimal surgical approach (Fig. 3):

1. If the RBH exceeds 6 mm: the transcrestal approach is the more favorable alternative because it is minimally invasive, and its morbidity, duration, and cost are limited.
2. If the RBH is between 4 and 6 mm, the transcrestal approach is the more favorable alternative when the sinus and membrane are in a relatively healthy state, which is the most common situations, whereas the lateral approach is preferred when the sinus and membrane are in an unhealthy state. The RBH does not appear to affect osteogenesis, indicating that surgical difficulty and complications should be considered rather than osteogenesis in this situation. The presence of sinus diseases might have an important effect on the tensile potential of Schneiderian membrane, thus increasing the difficulty of surgery and the risk of postoperative complications.
3. If the RBH is between 2 and 4 mm, the lateral approach is the more favorable alternative when the sinus wall is less than 2 mm, which is the most common situations, otherwise, the transcrestal approach should be chosen. Membrane elevation requires good vision and access, and the incidence of membrane perforation correlates with the thickness of the maxillary sinus lateral wall. Thus, when accessing the antral cavity from a lateral wall more than 2 mm away, the transcrestal approach is more favorable alternative. Health state of Schneiderian membrane and sinus should also be considered when necessary.
4. If the RBH is less than 2 mm, we suggest that the stretching potential of Schneiderian membrane and risk of the surgery should be assessed comprehensively (Table 1). Histologically, the smaller the width of the maxillary sinus, the higher the level of osteogenesis can be anticipated after MSFA. Surgically, during elevation, the membrane perforation risk is increased by the presence of a narrow and tapered maxillary sinus cavity; however, the wider the floor of the maxillary sinus floor, the greater local tension stretches when lifting the maxillary sinus membrane. If a patient has a healthy maxillary sinus, and the maxillary contours tends to be oval and no septa are present in the lifting region, the stretching potential of the maxillary sinus membrane is relatively favorable (Type A: SFT). For a stretch-favorable case, the transcrestal approach is the more favorable alternative. If a patient has an unhealthy maxillary sinus, or the maxillary contours tend to be wide (or too sharp) and there is a septum within the lifting region, the stretching potential of the maxillary sinus membrane is relatively unfavorable (Type B: SUT), and the surgical difficulty and risks are relatively high. For a stretch-unfavorable cases, the lateral approach is recommended.

### Clinical cases

#### Case 1

**Fig. 4:**
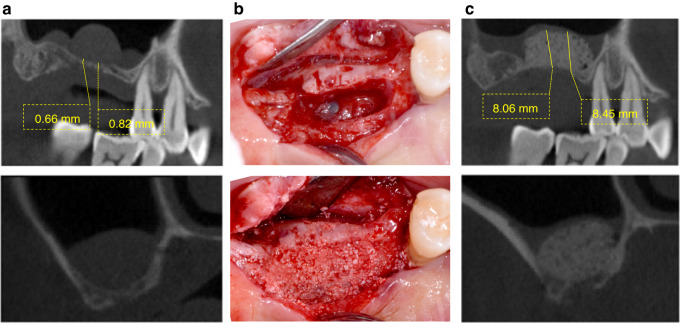
Case 1: **a** Pre-operative CBCT assessment, **b** surgical procedure, and **c** post-operative CBCT assessment of patient in case 1

This case comprised a 67-year-old male patient with a pseudocyst on the right maxillary sinus antral floor. Clinical examination revealed an edentulous maxilla encompassing the region from right first molar to the second molar. Assessment using pre-operative Cone Beam Computed Tomography (CBCT) revealed an atrophied edentulous ridge with an RBH < 1–2 mm (Fig. 4a). The maxillary contours tended to be oval and a homogeneous radiopaque mass without clinical symptoms was observed on the antral floor. The stretching potential of the maxillary sinus membrane was assessed as relatively favorable (SFT). Intentional perforation of the sinus membrane was carried out and a fine needle was used to aspirate the fluid to reduce the volume of the pseudocyst. Following saline irrigation, the transcrestal approach was used to elevate the sinus membrane. Subsequently, anorganic bone graft (Bio-Oss, Geistlich Pharma, Switzerland) was inserted (Fig. 4b). Post-operative CBCT showed that the sinus membrane was elevated and the bone graft material was successfully emplaced (Fig. 4c).

#### Case 2

**Fig. 5:**
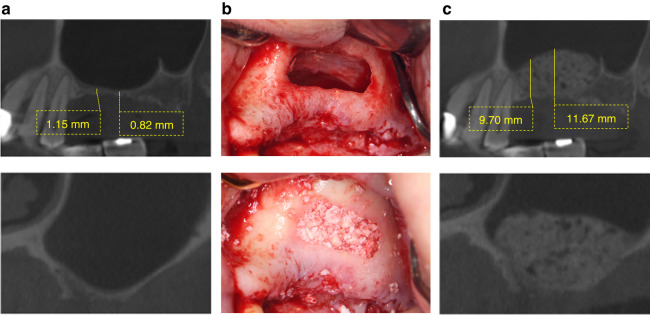
Case 2: **a** Pre-operative CBCT assessment, **b** surgical procedure, and **c** post-operative CBCT assessment of patient in case 2

This case was a 49-year-old female without sinus pathology. Clinical examination revealed an edentulous maxilla encompassing the region from the left first molar to the second molar. Assessment using pre-operative Cone Beam Computed Tomography (CBCT) revealed an atrophied edentulous ridge with an RBH < 1–2 mm (Fig. 5a). The maxillary sinus of the patient was in a healthy state, while the maxillary contours tended to be wide and there was a maxillary sinus septum in the distal part. The stretching potential of the maxillary sinus membrane was assessed as relatively unfavorable (SUT), and the surgical difficulty and risks were relatively high. The lateral wall approach was used to elevate the sinus membrane (Fig. 5b). Subsequently, the sinus cavity was compacted using an anorganic bone graft (Bio-Oss, Geistlich Pharma, Switzerland). Post-operative CBCT showed that the sinus membrane was elevated and the bone graft material was successfully emplaced (Fig. 5c).

## Discussion

Sinus pneumatization and ridge atrophy represent challenges to the successful rehabilitation of patients with posterior maxilla endosseous implants. Elevating the sinus comprises forming a “mucoperiosteal-alveolar pocket” above the maxillary floor and underneath the Schneiderian membrane to increase alveolar bone height and create a “confined space”.^18^

Although both osteotome and lateral window procedures are safe and effective in atrophic posterior maxilla, residual bone height is crucial in determining the survival of these implants,^89^ and sinus graft surgical decisions are majorly influenced by the RBH.^90–92^ With the improvement of implant surface modification and surgical equipment, the choice of MSFA approaches has been continuously updated and whether to choose immediate deferred placement of implants with anatomical variations is still controversial. No significant influence of RBH on the stability of the primary implant in atrophic sinus floor were reported, while initial RBH of less than 4 mm was reported to influence the success rates of implants inserted in combination with sinus floor elevation using osteotomes.^37–39^ Sinus elevation through the transcrestal window approach for a patient whose posterior maxilla had only 1–2 mm of residual alveolar bone was reported recently, and the incisions used in transcrestal window approach were shorter, compared with the lateral window approach, which could reduce discomfort of the patient after sinus elevation surgery.^11,93^ The evidence is insufficient and further long-term follow-up studies were needed. Meanwhile the transcrestal window approach requires a thorough assessment of the anatomy of sinus, elasticity, and deformation capacity of the Schneiderian membrane, the location of the intraosseous artery(which could be undetectable in CT/CBCT images), precise surgical approach, and so on.^93^ In addition, the crestal approach was used to elevate the sinus floor of 27 patients with residual bone heights of 2–3 mm.^94^ Moreover, a recent study revealed that the RBH and vital bone formation were not statistically correlated.^13^ No significant differences in the amount of osteogenesis in sinuses classified as having an RBH ≤ 2 mm or >2 mm were observed.^13^ When choosing the surgical technique, clinicians should assess the lateral wall thickness. A difference in the perforation rate was noted for a wall thickness measured at 6 mm coronal to the sinus floor.^21^ While other researchers also reported that lateral wall thickness had no effect on the perforation rate.^95^

Despite the predictability of sinus lift procedures, intra-operative complications are common,^96,97^ especially Schneiderian membrane perforation.^24,98–100^ Sinus compliance comprises the intrinsic potential of the sinus mucosa to resume its homeostatic status after the surgical trauma caused by sinus lifting.^18^ A higher rate of perforation is associated with a thinner membrane, possibly because the tensile capacity of a thicker membrane is significantly higher.^19^ Sinus augmentation surgery can be carried out on a 1–2 mm thick healthy and resilient membrane; however, for a thin membrane (<1 mm), a more cautious approach should be adopted.^65^ The Schneiderian membrane has the potential to thicken during inflammation, such as during odontogenic infections, especially apical infections.^63^ Irritations, such as allergies, can also thicken the mucosa.^59^ However, sinus augmentation is not contraindicated by the presence of mild mucosal thickening or pseudocysts in the absence of coexisting sinonasal symptoms.^101^ However, with a deeper understanding of the maxillary sinus disease, some researchers formed different opinions,^23^ and diseases of the maxillary sinus should be diagnosed and managed carefully prior to sinus elevating surgery.^96^

The stretching potential of Schneider’s membrane should be considered surgically and histologically.^13,33^ After lateral sinus floor elevation surgery, transient swelling of sinus membrane is observed, which reaching a peak value 7 days after surgery and completely resolves over months.^62^ The widths and contours of the sinus are closely related. For example, Wang et al. described the palate-nasal-recess (PNR) as the intersection point of the two imaginary lines following the lower part of the lateral nasal wall and the palatal wall in the maxillary sinus,^102^ which would make elevation of the sinus membrane more complicated and enhance the risk of membrane perforation. Niu et al. considered the sinus width, sinus depth, the PNR, and angle A simultaneously.^33^ A flat or irregular maxillary sinus stretches more when lifted to the same height, which requires a better stretch potential of the Schneider membrane. For example, the presence of irregular bone walls, exostosis, and septa might contribute to the occurrence of sinus perforations.^87^ Perforation rates of MSFA when septa were present were much higher than in those without septa.^86,87^ However, at 6–9 months post-surgery, wider sinuses added with deproteinized bovine bone mineral (DBBM) alone showed a lower percentage of newly formed bone, while the sinus contours and the RBH and sinus contours did not appear to influence post-MSFA osteogenesis.^13^

## Conclusion

Anatomical factors, including the remaining alveolar bone, maxillary sinus characteristics, the health condition of the Schneiderian membrane, and the lateral wall thickness, crucially affect successful lifting. The stretching potential affected by maxillary sinus characteristics and the health condition of Schneiderian membrane/sinus, as well as the residual bone height, influence clinical outcomes and the choice of surgical techniques, which should be taken into account by clinicians.
